# Computational identification of natural inhibitors targeting GroEL in *Leptospira interrogans*: an integrative virtual screening and molecular dynamics approach

**DOI:** 10.3389/fcimb.2025.1733096

**Published:** 2026-02-02

**Authors:** Guneswar Sethi, Sthitaprajna Sahoo, Su-Cheol Han, Donghyun Shin, Jeong Ho Hwang

**Affiliations:** 1 Center for Large Animals Convergence Research, Korea Institute of Toxicology, Jeongeup-si, Jeollabuk-do, Republic of Korea; 2 Department of Agricultural Convergence Technology, Jeonbuk National University, Jeonju, Republic of Korea; 3 Division of Advanced Predictive Research, Center for Bio-Signal Research, Korea Institute of Toxicology, Daejeon, Republic of Korea

**Keywords:** density functional theory, free energy landscape, leptospirosis, molecular dynamics simulation, principal component, structure-based virtual screening

## Abstract

**Introduction:**

Leptospirosis is a zoonotic disease caused by *Leptospira interrogans* and represents a major public health and veterinary concern. The persistence of the pathogen is closely associated with biofilm formation, yet targeted therapeutics are currently unavailable. The GroEL chaperonin, a conserved protein involved in biofilm formation and immunogenicity, was investigated as a potential therapeutic target.

**Methods:**

A structure-based virtual screening approach was performed using a library of 543,503 natural compounds from the Life Chemicals database. Top-ranked ligands were evaluated using molecular docking and physicochemical and pharmacokinetic property analyses. Density functional theory calculations were performed to assess electronic stability, followed by molecular dynamics simulations to evaluate ligand–protein complex stability. Principal component analysis and MM-PBSA binding free energy calculations were subsequently applied to characterize conformational dynamics and binding affinity.

**Results:**

Five compounds (F3385-2019, F1243-0200, F3139-0927, F2801-0179, and F1864-0208) exhibited strong binding affinities toward GroEL, with docking energies ranging from −10.34 to −8.26 kcal/mol. All shortlisted compounds complied with Lipinski’s Rule of Five and demonstrated favorable pharmacokinetic properties. Molecular dynamics simulations and MM-PBSA analyses indicated stable ligand–protein interactions. Among the candidates, F1864–0208 and F1243–0200 emerged as the most stable and promising leads, whereas the remaining compounds showed moderate inhibition.

**Discussion:**

This study provides computational evidence supporting GroEL as a viable drug target in *L. interrogans*. The identified natural compounds may represent promising scaffolds for the development of novel anti-leptospiral agents. Further *in vitro* and *in vivo* studies are required to validate their therapeutic efficacy and safety.

## 1 Introduction

Leptospirosis, a neglected and re-emerging zoonotic infection caused by pathogenic spirochetes of the genus *Leptospira*, is a major global health concern, with *L. interrogans* identified as the most clinically significant species (Rajapakse et al., 2025). Its burden is particularly pronounced in tropical and subtropical regions, where environmental conditions and socioeconomic inequities drive its widespread transmission. The World Health Organization (WHO) estimates that leptospirosis affects over one million individuals annually, resulting in approximately 60,000 deaths, with the highest burden observed in low- and middle-income countries (LMICs) across Southeast Asia, Latin America, and Oceania (Douchet et al., 2022; Gizamba and Mugisha, 2023; Rajapakse et al., 2025). In developed regions, leptospirosis is increasingly categorized as an emerging infectious disease that is often linked to environmental exposure and international travel. Transmission occurs primarily through direct contact with the urine of infected animals or contaminated water, particularly under warm and humid conditions. Once in the host, *Leptospira* disseminates via the bloodstream, targeting organs such as the kidneys, liver, and lungs (Amamura et al., 2025). The ability of the pathogen to persist in renal tubules leads to prolonged urinary shedding, contributing to environmental contamination and continued transmission (Evangelista and Coburn, 2010). Domestic dogs, as key reservoirs, further amplify the zoonotic risk due to their prolonged shedding and close contact with humans (Azócar-Aedo and Monti, 2022; Guzmán et al., 2023).

Currently, leptospirosis is treated with broad-spectrum antibiotics such as doxycycline, penicillin, and third-generation cephalosporins (Mendu et al., 2025). However, the treatment results vary in severe cases. New reports on antibiotic resistance have raised concerns about its long-term effectiveness. Furthermore, existing vaccines are limited and only protect against certain serovars. Therefore, there is an urgent need to identify novel and specific drug targets for the treatment of leptospirosis. A major challenge in controlling leptospirosis is the ability of the pathogen to form biofilms, which confer resistance to immune defenses and antibiotics (Davignon et al., 2023, 2024). Biofilm formation, which is observed in both laboratory and natural settings, plays a crucial role in the pathogen’s persistence and transmission (Dias and Pinna, 2025). The molecular mechanisms underlying biofilm formation remain incompletely understood but likely involve bacterial stress responses and proteins such as GroEL (Vinod Kumar et al., 2017).

GroEL, a member of the highly conserved HSP60 family of molecular chaperones, plays a central role in bacterial proteostasis by facilitating proper folding of nascent and stress-denatured proteins (Ho et al., 2021; Singh et al., 2024). In *Leptospira interrogans*, GroEL is essential for survival under host-induced stress conditions, such as elevated temperatures and oxidative damage, thereby significantly contributing to the pathogen’s virulence, biofilm formation, and environmental persistence (Ho et al., 2021). Beyond its chaperone function, GroEL mediates adhesion to host tissues and induces the release of proinflammatory cytokines, underscoring its involvement in pathogen-host interactions (Ho et al., 2021). GroEL is also highly immunogenic and has been detected in the sera of patients with leptospirosis (Vinod Kumar et al., 2017). In contrast to other leptospiral antigens, such as LigA and LipL32, which exhibit antigenic variability and limited protective efficacy (Lucas et al., 2011), GroEL is evolutionarily conserved and indispensable for bacterial viability across diverse species. Disruption of GroEL function has been shown to impair protein homeostasis, stress tolerance, and cellular survival, resulting in defective bacterial growth (Fayet et al., 1989; Taguchi and Koike-Takeshita, 2023; Wang et al., 2025). Consistently, experimental and pharmacological studies demonstrate that small-molecule inhibition of GroEL suppresses bacterial proliferation and virulence in both Gram-positive and Gram-negative pathogens, confirming its suitability as a druggable target (Abdeen et al., 2016; Godek et al., 2024; Zhang et al., 2025). Collectively, these findings provide a strong biological and experimental rationale for prioritizing GroEL as a target for structure-based inhibitor discovery in the development of anti-leptospiral drugs.

In this context, computational drug discovery has emerged as an efficient strategy for the rapid identification of antibacterial leads through structure-based modeling, stability evaluation, and binding-energy analysis (Sadybekov and Katritch, 2023). These approaches have increasingly been applied to natural-product–derived antibacterial agents; for instance, Verma et al. employed structure-based virtual screening of *Allium sativum* phytocompounds to identify novel antimicrobial candidates (Verma et al., 2024). Within this framework, targeting GroEL using this approach is particularly promising, and natural products provide an excellent source of candidates because of their unique scaffolds, proven bioactivity, and generally favorable safety profiles. In this study, we employed a comprehensive *in silico* drug discovery pipeline to identify natural product-based inhibitors that target GroEL in *L. interrogans*. A structurally diverse library of natural compounds was screened against GroEL using structure-based virtual screening (SBVS), followed by detailed binding affinity and interaction profiling of the top-ranked ligands. The electronic and chemical characteristics of the ligands were mapped using density functional theory (DFT). ADME property prediction was used to evaluate drug-likeness, oral bioavailability, and pharmacokinetic profiles. In addition, molecular dynamics simulations (MDS) were performed to analyze the structural stability and dynamic behavior of the selected complexes, whereas principal component analysis (PCA) was used to investigate the essential motions of GroEL and the impact of ligand binding on its conformational landscape. Binding free energy estimations were performed using the MM-PBSA method to assess the thermodynamic favorability of the protein–ligand interactions.

Overall, this integrated computational strategy enabled the identification of natural product-based ligands with high binding affinities, favorable pharmacokinetic properties, and the ability to stabilize the GroEL structure. These findings provide a strong foundation for future experimental validation and highlight the therapeutic potential of targeting GroEL in the development of novel treatments for leptospirosis.

## 2 Materials and methods

### 2.1 GroEL structure and active site prediction

**Figure 1:**
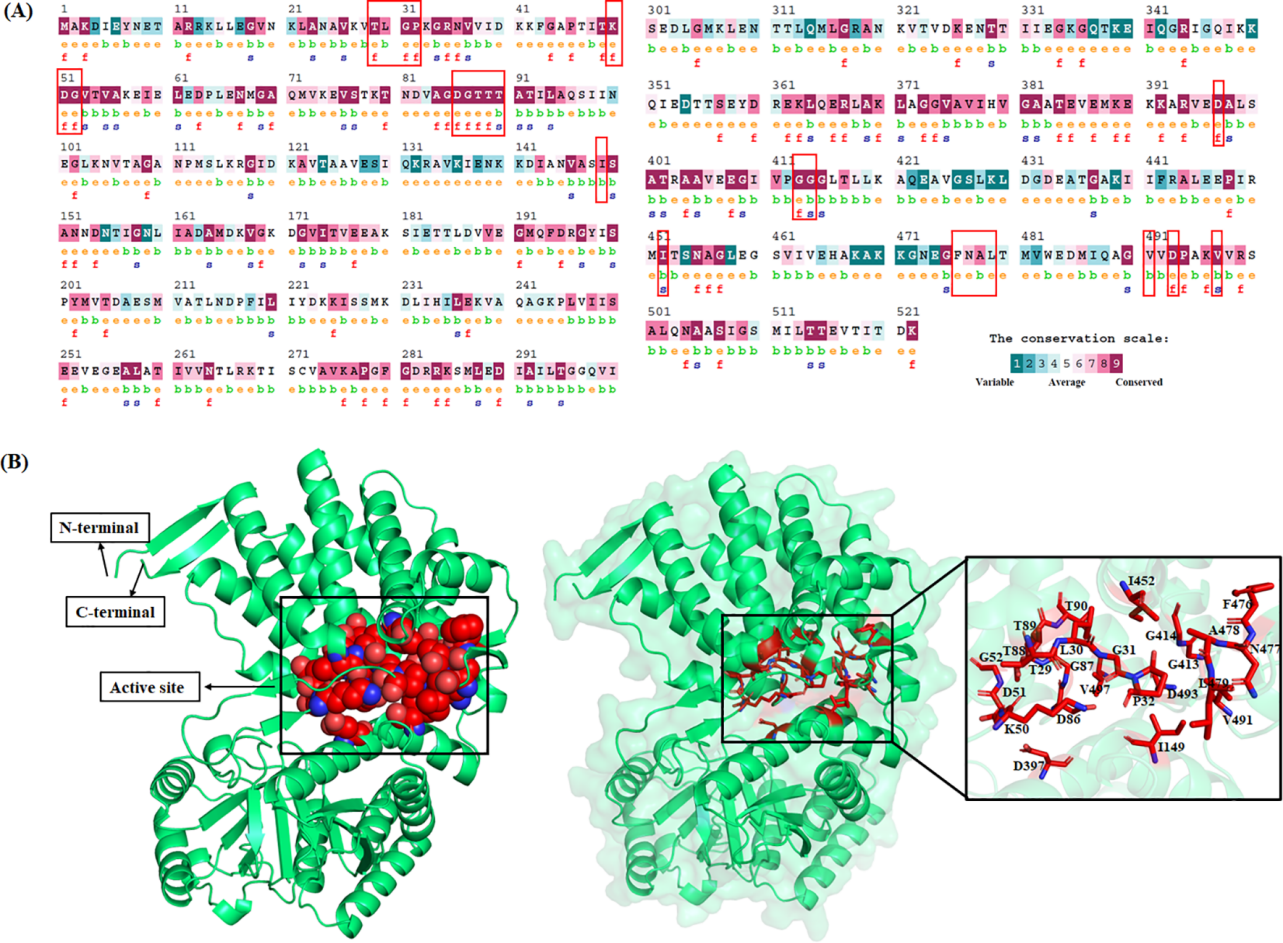
Sequence conservation and structural features of GroEL from *Leptospira interrogans*. **(A)** Residue-wise evolutionary conservation profile of GroEL based on multiple sequence alignment, with predicted active-site residues highlighted in red boxes. **(B)** Three-dimensional structure of GroEL showing the overall fold, N- and C-terminal domains, and the active-site region highlighted in red, with an enlarged view of key binding-pocket residues.

The sequence of the GroEL protein (P61439), which comprises a total of 546 amino acids, was retrieved from the UniProt database in FASTA format (The UniProt Consortium, 2017). Subsequently, the 3D structure of GroEL was predicted using the AlphaFold program (Jumper et al., 2021) (**Figure 1**). To identify potential active site residues in GroEL, six complementary structure-based prediction tools were employed: COACH (Yang et al., 2013), TM-SITE (Yang et al., 2013), S-SITE, COFACTOR (Roy et al., 2012), FINDSITE (Brylinski and Skolnick, 2008), ConCavity (Capra et al., 2009), and CASTP (Tian et al., 2018). The outputs of these tools were used to generate a consensus list of residues for subsequent virtual screening experiments.

### 2.2 Structure-based virtual screening

**Figure 2:**
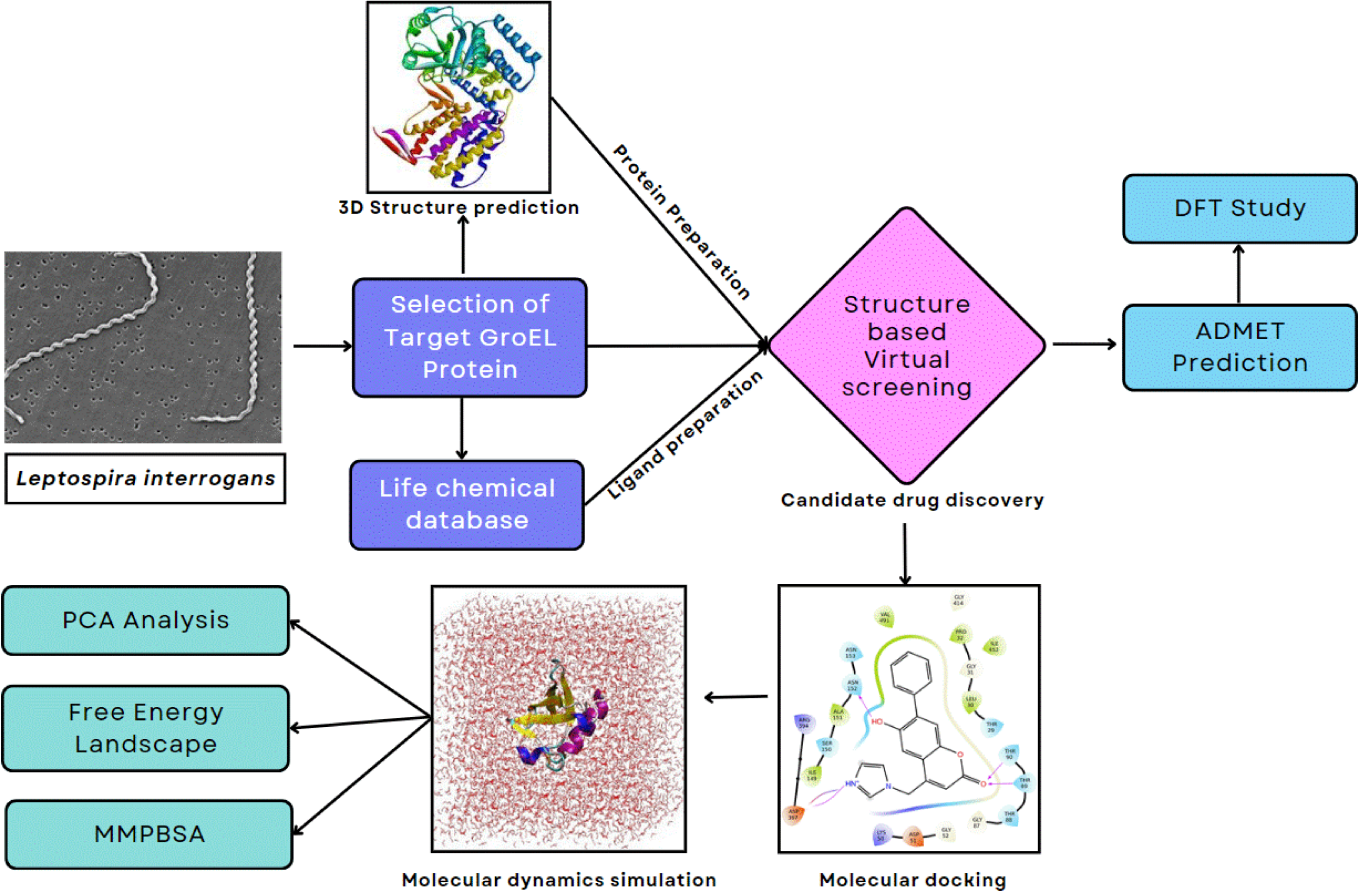
Conceptual overview of the structure-based virtual screening and post-screening computational analyses applied for the discovery of potential GroEL inhibitors.

Virtual screening is a computational method that uses high-performance computing systems to identify, filter, and evaluate potential molecular conformations of chemical compounds from extensive databases. The structure of GroEL was prepared for virtual screening using the Protein Preparation Wizard in the Schrödinger Suite (Schrödinger, LLC, New York, NY, 2017-1) (Sahoo et al., 2024a; Sethi et al., 2024). The preparation steps included reconstructing the missing side chains and loops, assigning bond orders, adding hydrogen atoms at neutral pH, and removing non-essential water molecules. The structure was then subjected to energy minimization to resolve steric clashes and optimize the geometry. A compound library of 543,503 small molecules was obtained from the Life Chemicals Database. The ligands were processed using the LigPrep module of the Schrödinger Suite to generate low-energy conformations, assign ionization states, and optimize geometries. Docking grids were created around the predicted active-site residues of GroEL. SBVS was conducted using the GLIDE module. Three levels of precision were employed sequentially: High-Throughput Virtual Screening (HTVS) for rapid initial screening, Standard Precision (SP) for refinement, and Extra Precision (XP) for detailed ligand–receptor interaction assessment. The top-ranking compounds from XP docking were retained for further analysis. Molecular interactions and binding poses were visualized using UCSF Chimera (Pettersen et al., 2004) and the GLIDE 2D interaction diagram tool. **Figure 2** presents the overall computational workflow adopted for the structure-based virtual screening of GroEL.

### 2.3 Drug-likeness analysis

The absorption, distribution, metabolism, and excretion (ADME) properties of all selected chemical scaffolds were evaluated using the QikProp module from the Schrödinger Suite (Schrödinger, LLC, New York, NY, 2017-1). This extensive assessment provides valuable insights into their potential as drug candidates by examining essential pharmacokinetic parameters. Key molecular descriptors were considered, including compliance with the Rule of Five (Ro5) (Lipinski et al., 1997). Together, these evaluations provided an integrated understanding of both the pharmacokinetic behavior and potential safety liabilities, thereby supporting a more reliable assessment of the drug-like properties of the compounds for subsequent studies.

### 2.4 Frontier molecular orbital analysis

Quantum mechanical calculations were performed using DFT to investigate the electronic characteristics and geometric configurations of the target compounds using the Jaguar module of the Schrödinger Suite (version 8.7) (Bochevarov et al., 2013). This computational approach provides an efficient and accurate framework for determining molecular electronic structures, optimized geometries, and physicochemical properties at the ground state level. The calculations focused on identifying critical electronic descriptors, including the highest occupied molecular orbital (HOMO), lowest unoccupied molecular orbital (LUMO), HOMO-LUMO energy gap (ΔE), and molecular electrostatic potential (MEP) surfaces. All of the computations employed the B3LYP hybrid density functional in conjunction with the 6-31++G(d,p) basis set (Lee et al., 1988; Bhatta et al., 2015). These comprehensive analyses provided valuable insights into the electronic reactivity, charge distribution, and potential binding characteristics of the designed molecules.

### 2.5 Molecular dynamics simulation

MDS were conducted to examine the structural stability and dynamic behavior of the top-ranked protein-ligand complexes. Simulations were performed using the GROMACS 2022.3 software package with the CHARMM36 force field, which is widely recognized for its reliability in all-atom protein-ligand modeling (Bekker et al., 1993; Van Der Spoel et al., 2005). Ligand topologies were generated using the CGenFF server (Vanommeslaeghe et al., 2010) and integrated with the protein topology files prepared using the CHARMM36 force field (Bjelkmar et al., 2010). Each protein-ligand complex was positioned within a cubic simulation box, maintaining a 1nm buffer between the solute and box boundaries. The system was solvated using the TIP3P water model to simulate a realistic aqueous environment (Jorgensen et al., 1983). A two-step energy minimization process was performed to remove steric clashes and achieve an energetically favorable conformation. The steepest descent algorithm was first applied, followed by the conjugate gradient method, with 50,000 minimization steps. Following energy minimization, the system was equilibrated under both the NVT and NPT ensembles for 100 picoseconds (ps) each. Temperature was controlled using the Berendsen thermostat (Berendsen et al., 1984), and pressure was regulated using the Parrinello–Rahman barostat (Parrinello and Rahman, 1981). After the equilibration phase, MDS were executed for 100 ns for each protein-ligand complex. The coordinates of each complex were recorded at regular intervals of 2 fs. The final resulting trajectory files were analyzed using built-in GROMACS tools to assess the stability of the GroEL-ligand complexes. Parameters such as the root-mean-square deviation (RMSD), root-mean-square fluctuation (RMSF), radius of gyration (Rg), hydrogen bonding interactions, and solvent-accessible surface area (SASA) were examined to determine the dynamic behavior and conformational stability of the complexes throughout the simulation, as described in our previous studies (Sahoo et al., 2024b, 2025).

### 2.6 PCA-based free energy landscape analysis

PCA, a robust method for multivariate statistical analysis, was employed to identify and characterize the primary motions within the protein-ligand complexes. This approach involves calculating the dominant motions using eigenvalues and eigenvectors, as previously described (Sahoo et al., 2023; Samantaray et al., 2025). The analysis used the final 50 ns of the MDS trajectories to construct a covariance matrix, focusing on the backbone atoms of the protein in each complex. The cosine content for the leading eigenvectors was computed to evaluate simulation convergence, and eigenvalues were subsequently obtained (Amadei et al., 1993). FEL analysis was performed to visualize the energy minima and their corresponding conformational states, allowing the identification of stable and metastable regions within the protein–ligand complexes (Maisuradze and Leitner, 2007). This approach provides insight into the system’s response to ligand binding by elucidating the underlying energetics and quantifying the overall thermodynamic stability of the complexes. The FEL was constructed using the first two principal components (PC1 and PC2), obtained from PCA, using the *g_sham* module in GROMACS (Lindahl et al., 2001). The resulting FEL plots were generated and visualized using Mathematica software.

### 2.7 MM-PBSA based binding affinity assessment

The Molecular Mechanics Poisson–Boltzmann Surface Area (MM-PBSA) method was employed to calculate the protein-ligand complex binding free energies (BFE) (Genheden and Ryde, 2015). The MM-PBSA is a widely used computational approach in drug design that combines molecular mechanics force fields, continuum solvent models, and solvation energy terms to estimate the thermodynamic properties of protein-ligand interactions. The BFE is derived from three main energy components: non-polar solvation energy, polar solvation energy, and vacuum potential energy. To assess the stability of the complexes, MDS were run for last 50 ns of the trajectory information, and the *g_mmpbsa* tool was used with default settings and a solute dielectric constant of 2.0 (Kumari et al., 2014). This method allows the decomposition of the binding free energy into various contributions, such as van der Waals interactions, electrostatic forces, solvation energy, and entropy, aiding in understanding the individual interactions that influence binding affinity. MM-PBSA calculations are critical for evaluating ligand binding affinities, comparing ligand poses, and optimizing drug candidates, and can be validated by experimental binding data to ensure the reliability of the computational predictions.

## 3 Results and discussion

### 3.1 Virtual screening and interaction mapping

Structure-based virtual screening (SBVS) remains a pivotal strategy in contemporary drug discovery, particularly for identifying novel inhibitors of key protein targets involved in pathogenic processes, such as biofilm formation (Oselusi et al., 2024). In the present study, SBVS was employed to explore potential inhibitors targeting the GroEL protein of *Leptospira interrogans*, a molecular chaperone known to be involved in biofilm development and host-pathogen interactions. Active site prediction tools collectively identified 26 candidate residues, including T29, L30, G31, P32, D86, G87, T88, T89, T90, S149, G413, and G414. Several residues were consistently predicted by all six methods, reinforcing their reliability. A comparison with previously reported conserved regions revealed a strong overlap. Specifically, residues T29–P32 were located near the apical domain (191–202), suggesting a role in substrate recognition; residues D86–S149 corresponded to the intermediate domain (residues 362–381), associated with conformational flexibility, and residues G413–G414 were mapped to the equatorial domain (401–415), a region that is essential for ATP hydrolysis and chaperone activity (Ho et al., 2021). The residual evolutionary conservation profile of GroEL, highlighting the active site residues and conserved variants considered for MD simulation, is illustrated in **Figure 2A** and **Figure 2B**. A compound library consisting of 543,503 small molecules from the Life Chemicals database was screened against the GroEL active site. The screening protocol utilized hierarchical docking within the GLIDE module, progressively filtering candidates using HTVS, SP, and XP protocols. This workflow enabled the systematic enrichment of compounds with high binding potential at the predicted active site of GroEL. The multi-step screening pipeline successfully reduced the compound library to the top 10% of molecules with the most favorable binding scores. Structural visualization highlighted key binding residues within the predicted GroEL active site. In particular, residues G413 and G414, which were identified by all prediction tools and located within a conserved functional domain, emerged as potential anchoring sites for ligand binding.

**Figure 3:**
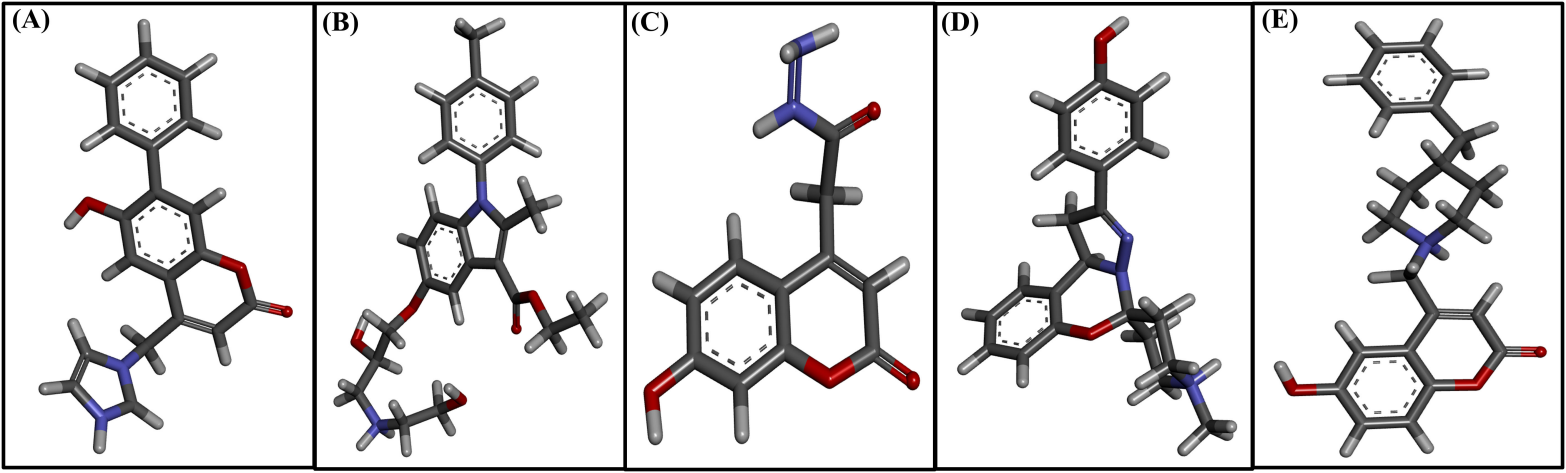
Three-dimensional molecular representations of the highest-ranking compounds showing their spatial conformations and orientation within the binding pocket. **(A)** F3385-2019, **(B)** F1243-0200, **(C)** F3139-0927, **(D)** F2801-0179, and **(E)** F1864-0208.

**Figure 4:**
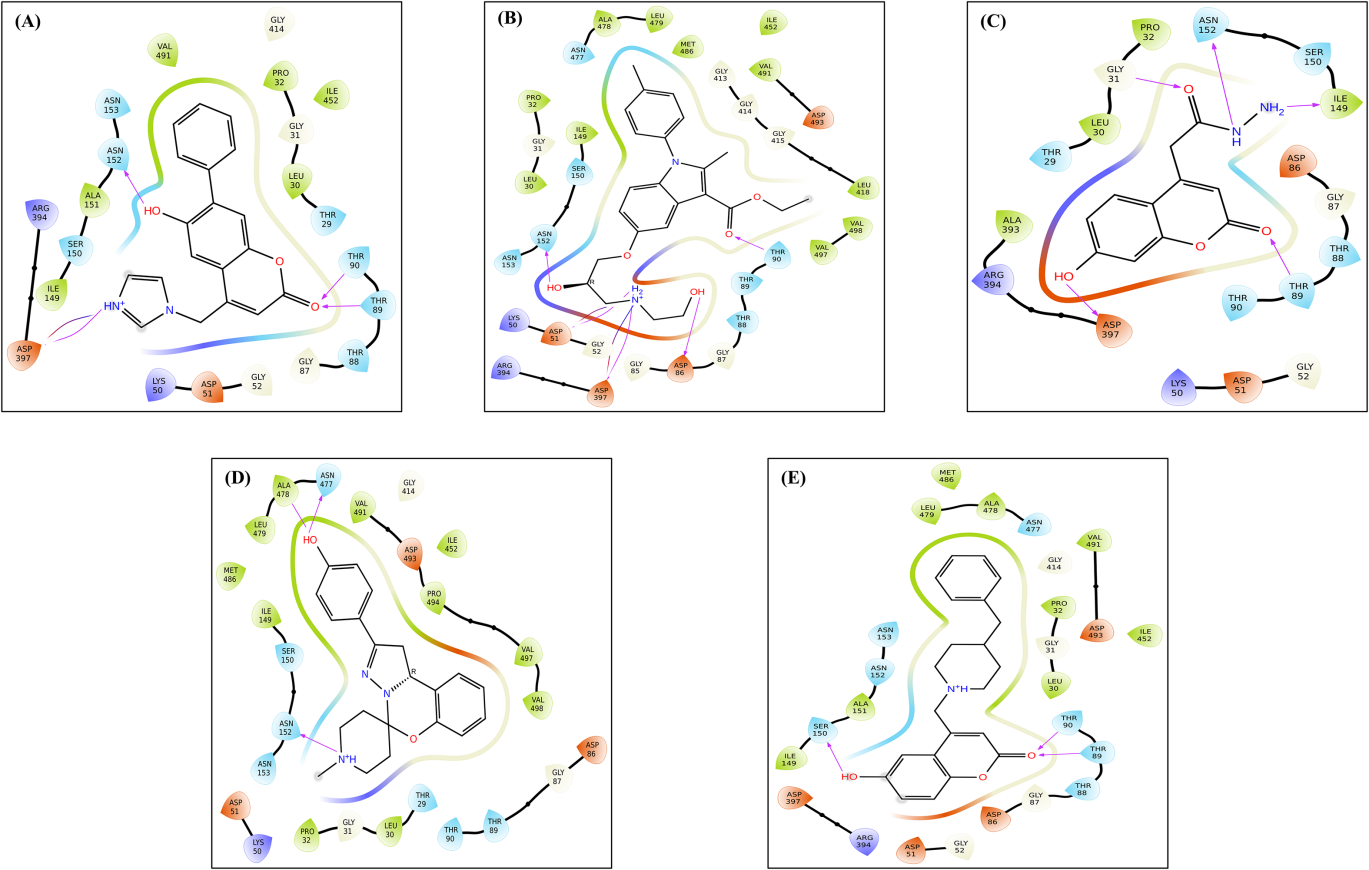
Molecular interaction diagrams for the lead compounds **(A)** F3385-2019, **(B)** F1243-0200, **(C)** F3139-0927, **(D)** F2801-0179, and **(E)** F1864-0208. Salt bridges are depicted using blue or red lines, whereas hydrogen bonding is illustrated with purple arrows. Amino acid residues are color-coded based on their properties: polar residues (light blue), acidic residues (orange), basic residues (blue), and nonpolar residues (green).

Following the XP docking stage, five lead compounds F3385-2019, F1243-0200, F3139-0927, F2801-0179, and F1864–0208 were shortlisted based on their favorable docking scores (all less than -8.0 kcal/mol), indicating strong binding potential and high affinity for the GroEL binding site (**Table 1**). Among these, F3385–2019 exhibited the most favorable binding characteristics, achieving a docking score of −10.343 kcal/mol. F3385–2019 formed three hydrogen bonds with the key residues Thr90, Asn152, and Asp397; and establishes hydrophobic contacts with Leu30, Gly31, Pro32, Lys50, Asp51, Thr89, Gly87, Ile149, and Ser150, indicating a robust and well-anchored interaction within the active site of the GroEL protein. Compound F1243–0200 closely followed a docking score of −9.668 kcal/mol, stabilized by four hydrogen bonds involving Asp86, Gly87, Thr90, and Asn152. Its hydrophobic interaction profile was extensive, involving multiple conserved residues, including Gly31, Lys50, Leu30, Pro32, Asp493, Val491, and others, suggesting deep and stable accommodation within the binding pocket. F3139–0927 achieved a docking score of −8.445 kcal/mol, forming hydrogen bonds with Gly31, Gly87, Thr88, and Ile149, and hydrophobic interactions with Arg394, Lys50, Asp86, and Leu30, indicating moderate but potentially meaningful binding. Similarly, F2801–0179 demonstrated a docking score of −8.364 kcal/mol and was stabilized through hydrogen bonding with Asn152 and Ala478, supported by hydrophobic contacts involving residues like Thr29, Gly87, Ile149, and Val491, contributing to favorable ligand positioning and binding strength. Finally, F1864–0208 displayed a docking score of −8.257 kcal/mol. Despite being the lowest among the top five, it formed two hydrogen bonds with Thr89 and Thr90 and engaged in extensive hydrophobic interactions with key residues, including Asp493, Ser150, Asp86, Gly414, and Asn477, thereby retaining its binding favorability. Overall, detailed molecular interaction analyses suggested that these five lead compounds effectively interacted with GroEL, with F3385–2019 being the top candidate. These findings reinforce the potential of these small molecules as inhibitors of GroEL-mediated biofilm formation by *Leptospira interrogans*. The two-dimensional chemical architectures of the identified scaffold compounds are shown in **Figure 3**. The binding interactions were systematically characterized, with particular emphasis on hydrogen bond networks, hydrophobic contacts, and π-π stacking arrangements that contribute to complex stabilization (**Figure 4**).

**Table 1:** Comparative assessment of molecular interaction patterns between shortlisted natural compounds and the target protein based on virtual screening results.

Si. no.	Compounds	Molecular weight (Da)	Glide docking score (kcal/mol)	Hydrogen bond interaction	Hydrophobic interaction
1	F3385-2019	318.33	–10.343	Thr90, Asn152, Asp397	Leu30, Gly31, Pro32, Lys50, Asp51, Thr89, Gly87, Ile149, Ser150
2	F1243-0200	426.51	–9.668	Asp86, Gly87, Thr90, Asn152	Gly31, Lys50, Asp51, Thr88, Leu30, Pro32, Asp397, Ser150, Ile 149, Val498, Val497, Asp493, Gly414, Asn477, Val491
3	F3139-0927	234.21	–8.445	Gly31, Gly87, Thr88, Ile 149	Arg 394, Asn152, Lys50, Asp51, Thr29, Asp397, Asp86, Leu30, Leu30
4	F2801-0179	349.43	–8.364	Asn152, Ala478	Pro32, Gly31, Lys50, Thr29, Thr90, Leu30, Gly87, Ser150, Asp493, Asp86, Ile452, Ile149, Gly414, Val491, Asn477
5	F1864-0208	349.42	–8.257	Thr89, Thr90 (2)	Pro32, Asn 153, Asp493, Leu30, Gly87, Asp86, Asp51, Asp397, Thr88, Ser150, Ile149, Gly414, Asn477, Ala478, Val491

### 3.2 ADME and drug-likeness property prediction

Evaluation of ADME properties is essential for identifying potential drug candidates with optimal pharmacokinetic and safety profiles. In this study, the QikProp module of the Schrödinger Suite was used to predict the key ADME descriptors and drug-likeness characteristics of the top-ranking hit compounds (**Table 2**). These computational predictions provide preliminary insight into the oral bioavailability, permeability, and systemic exposure potential of the selected molecules. All five compounds were assessed for compliance with Ro5 criteria, which is a benchmark for drug-likeness prediction based on molecular weight, lipophilicity, and hydrogen bonding potential. Notably, none of the compounds violated the Ro5 criteria, indicating favorable physicochemical properties and supporting their potential as orally available drug-like molecules. The predicted human oral absorption (HOA) values were above 80% for all compounds except F3139-0927, which showed a comparatively lower value of 52.24%. Particularly, F2801–0179 demonstrated the highest HOA (96.10%), suggesting excellent oral absorption potential. The remaining compounds, including F3385–2019 and F1243-0200, also exhibited high HOA values of 90.91% and 82.31%, respectively. Membrane permeability predictions using MDCK (QPPMDCK) and Caco-2 (QPPCaCo) models indicated strong cellular permeability for most compounds. F2801–0179 exhibited the highest permeability, with QPPMDCK and QPPCaCo values of 300.27 and 573.84, respectively, followed by F3385-2019 (QPPMDCK: 214.34, QPPCaCo: 461.25), highlighting their efficient potential for intestinal and blood–brain barrier passage. The logP (QPlogPo/w) values ranged between −0.559 and 3.481, indicating acceptable lipophilicity for all compounds. Interestingly, F3139–0927 had a negative logP, suggesting higher hydrophilicity, which may explain its lower HOA and permeability values. The hydrogen bond donor and acceptor counts were also within favorable limits, ranging from 1–4 and 5.25–7.65, respectively. These values suggest that balanced polar surface characteristics are essential for bioavailability and target engagement. Collectively, the ADME predictions demonstrate that F2801–0179 and F3385–2019 possessed pharmacokinetic and drug-likeness profiles, combining high oral absorption, strong membrane permeability, and no violations of drug-likeness rules. These findings reinforce the need for further preclinical studies.

**Table 2:** Detailed summary of the predicted ADME properties of the top hit molecules using QikProp.

Compounds	MW	QPlogPo/w	QPlogBB	QPPMDCK	QPPCaCo	Donor HB	Accept HB	HOA	Rule of five violation
F3385-2019	318.33	2.78	–0.989	214.339	461.246	1	5.25	90.912	Nil
F1243-0200	426.51	3.481	–1.441	40.626	90.174	3	7.65	82.319	Nil
F3139-0927	234.21	–0.559	–1.824	15.037	39.476	4	6.25	52.243	Nil
F2801-0179	349.43	3.378	0.263	300.27	573.841	1	5.5	96.103	Nil
F1864-0208	349.42	3.289	–0.547	79.303	167.431	1	5.25	86.005	Nil

Molecular weight, in Da (130–725 Da).

QPlogPo/w: Predicted octanol/water partition coefficient (acceptable range: 2.0 to 6.5).

QPPMDCK: Predicted apparent MDCK cell permeability in nm/s (25 poor, > 500 great).

QPPCaCo: Predicted apparent CaCo-2 cell permeability in nm/s (< 25 poor, > 500 great).

QPlogBB: Predicted brain/blood partition coefficient. (−3.0 – 1.2).

Donor HB: No. H bonds donated by the molecule (range: 0–6).

Accept HB: No. H bonds accepted by the molecule (range: 2–20).

Percentage of human oral absorption (< 25% poor and > 80% is high).

### 3.3 Density functional theory analysis

**Figure 5:**
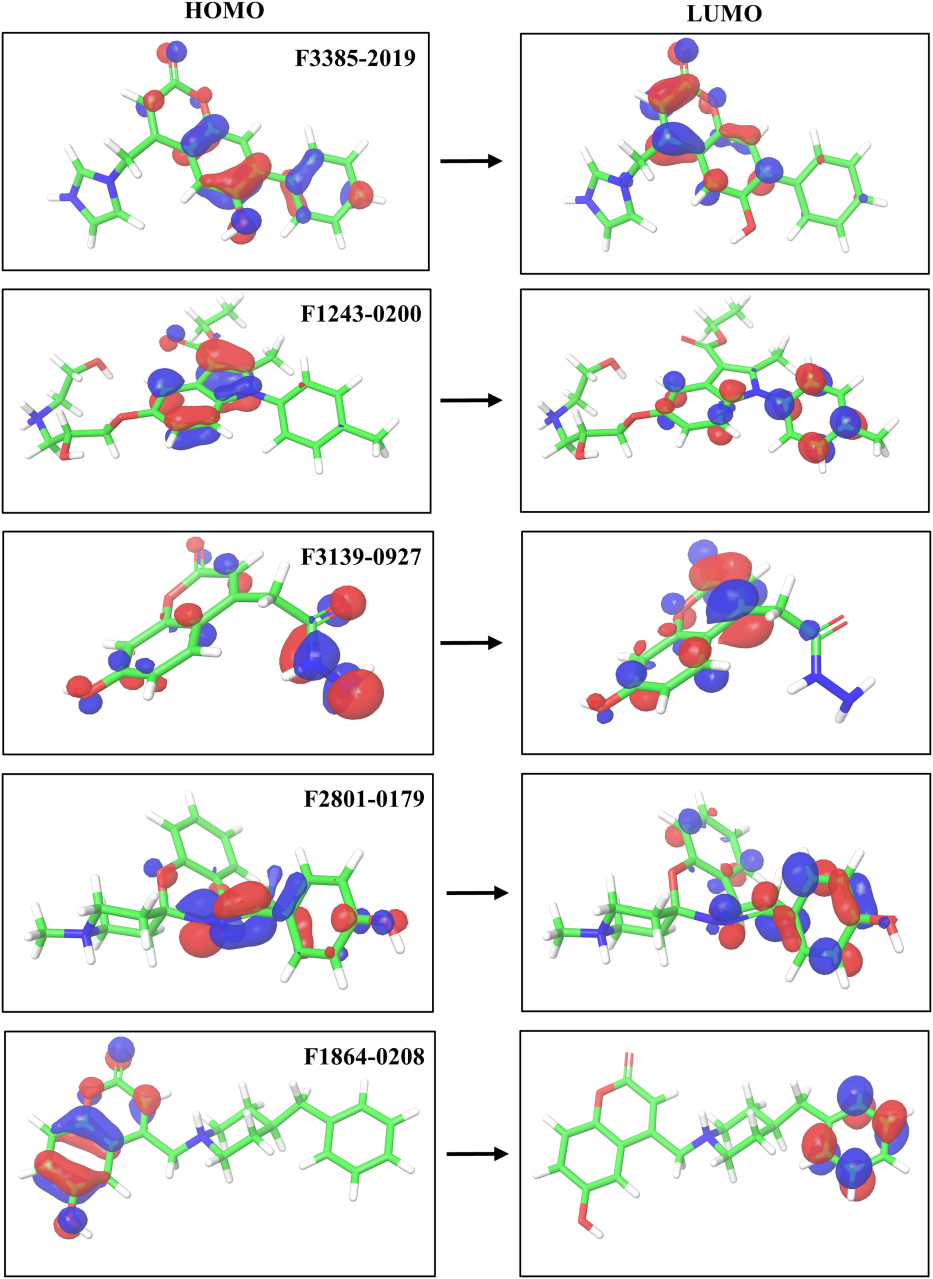
Electronic orbital analysis illustrates the spatial distribution of HOMO and LUMO for compounds F3385-2019, F1243-0200, F3139-0927, F2801-0179, and F1864-0208.

DFT calculations were performed using the Jaguar module of the Schrödinger Suite to assess the electronic characteristics and possible reactivities of the identified hit compounds. The HOMO and LUMO energies were determined, and the HOMO–LUMO energy gap (ΔE) was analyzed to evaluate the electronic stability and chemical reactivity of each compound (**Table 3**). Among the top candidates, F2801–0179 exhibited the narrowest HOMO–LUMO gap of −0.186 eV, suggesting high chemical reactivity and enhanced potential for electron transfer interactions with the GroEL protein. This was closely followed by F1243-0200, which showed a ΔE of −0.173 eV, and F3139–0927 with −0.164 eV, both indicative of favorable reactivity profiles. F3385–2019 showed a moderately wider gap of −0.141 eV, implying slightly lower reactivity but still within a range conducive to bioactivity. Interestingly, F1864–0208 displayed an anomalously large gap of −1.9 eV (**Figure 5**), which may either reflect a computational artifact or suggest a very low electron transfer potential, possibly correlating with its relatively lower docking scores. FMO analysis highlighted the electronic diversity among the top-ranked compounds and supported the findings of molecular docking analysis. Molecules with smaller energy gaps, particularly F2801-0179, are more likely to undergo charge transfer interactions with amino acid residues in the protein active site, thus enhancing binding strength and biological efficacy (**Figure 5**).

**Table 3:** Frontier molecular orbital (FMO) properties of identified top-hit molecules were analyzed using the Jaguar Module in Schrödinger Suite.

Si. no	Compounds	HOMO energy (eV)	LUMO energy (eV)	Energy gap (eV)
1	F3385-2019	–0.223	–0.082	–0.141
2	F1243-0200	–0.177	–0.004	–0.173
3	F3139-0927	–0.229	–0.065	–0.164
4	F2801-0179	–0.201	–0.015	–0.186
5	F1864-0208	–0.198	–0.008	–1.9

### 3.4 Structural stability analysis

**Figure 6:**
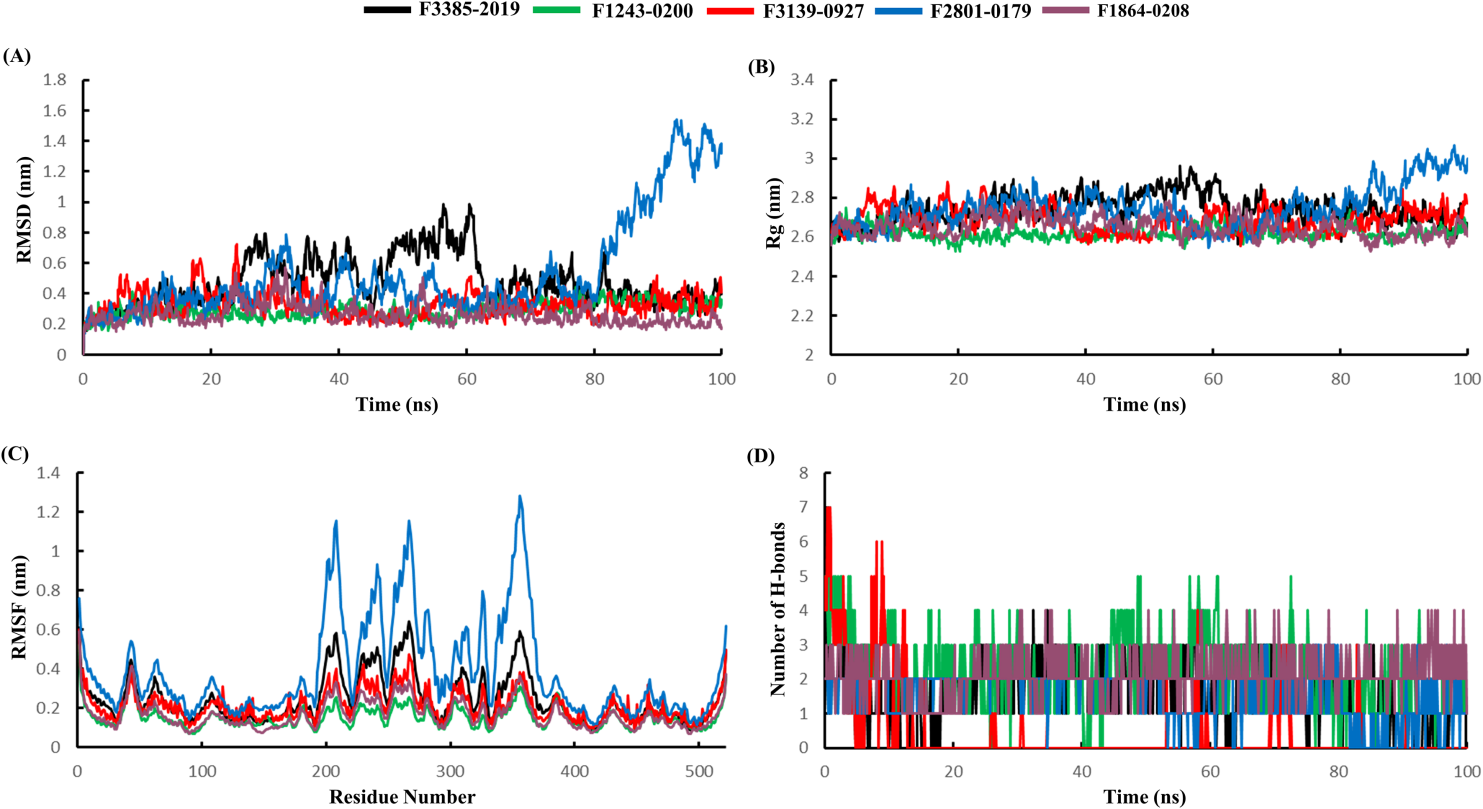
Molecular dynamics simulation (MDS) analysis illustrating the **(A)** Protein backbone RMSD, **(B)** Rg, **(C)** RMSF, and **(D)** H-bond analysis. Importantly, the reduced flexibility observed near functional residues may reflect strong protein–ligand interactions that limit local motion, particularly in F1243–0200 and F1864–0208 complexes, indicating their potential to maintain structural rigidity in binding pockets.

To reinforce the reliability of virtual screening outputs, MDS, PCA, and MM-PBSA free energy calculations have proven essential for validating structural stability and refining predicted binding affinities. Multiple studies demonstrate the utility of this approach; for example, MDS and MM-PBSA have been applied to prioritize repositioned inhibitors targeting PfEMP1 in *Plasmodium falciparum*, while docking, MDS, and experimental assays have been used to validate repurposed FDA-approved drugs against FZD10 in nasopharyngeal carcinoma (Ngernsombat et al., 2024; Verma et al., 2025). These findings underscore the importance of MD-based refinement as a critical step following virtual screening. To further elucidate the binding mechanism and stability of the selected natural ligands within the GroEL binding pocket, MD simulations were performed for 100 ns. The trajectories provided insights into the conformational behavior and ligand–protein interactions over the simulation timescale. Various structural and energetic parameters, including the RMSD, RMSF, Rg, hydrogen bond formation, SASA, and ligand RMSD, were assessed. The backbone RMSD trajectories (**Figure 6A**) provide a general view of the overall structural deviation and equilibration of GroEL upon ligand binding. Most ligand–protein complexes exhibited RMSD values stabilizing within 0.3 to 0.6 nm, indicative of good structural convergence. Notably, the F2801–0179 complex displayed higher backbone fluctuation during the final 30 ns of the simulation, with the RMSD peaking at approximately 1.7 nm, suggesting possible local conformational adjustments. In contrast, the F1243–0200 and F1864–0208 complexes exhibited relatively low and stable RMSD values throughout the simulation, maintaining fluctuations within 0.3 to 0.4 nm, indicating high structural integrity. F3385–2019 and F3139–0927 exhibited intermediate profiles, with moderate deviations (0.4–0.6 nm), suggesting favorable stability under dynamic conditions.

The Rg values, which reflect the overall compactness of the protein structure, remained consistent across all complexes (**Figure 6B**). The average Rg values ranged from 2.6 to 3.0 nm, indicating no significant unfolding or structural destabilization. However, the F2801–0179 complex showed a mild upward trend in Rg values after ~80 ns, correlating with its higher RMSD values and suggesting a slight expansion in structural volume. The remaining ligands, particularly F1243–0200 and F1864-0208, maintained steady Rg profiles, reinforcing the conclusion of a stable tertiary structure. RMSF analysis (**Figure 6C**) provided insight into residue-level fluctuations and local flexibility within the GroEL structure. The F2801–0179 complex exhibited elevated fluctuations across flexible loop regions, especially between residues 200–350, where peaks exceeded 1.2 nm, suggesting enhanced motion or flexibility in peripheral regions. In contrast, the F1243-0200, F1864-0208, and F3139–0927 complexes showed lower fluctuations (≤0.5 nm), particularly in regions critical for ligand binding, suggesting that these natural ligands effectively stabilize key domains of the protein. Hydrogen bonds play a crucial role in the stability and specificity of protein–ligand interactions. **Figure 6D** shows the number of hydrogen bonds formed between GroEL and each ligand over 100 ns. F1243–0200 consistently formed the highest number of hydrogen bonds and maintained up to six concurrent interactions throughout the simulation. This sustained H-bonding profile supports its strong affinity and anchoring capability. F1864–0208 also showed a stable H-bond pattern (2–4 bonds), whereas F2801–0179 and F3139–0927 exhibited more transient bonding, with counts fluctuating between 1 and 3. The F3385–2019 complex formed fewer but consistent hydrogen bonds (1–2), which may indicate hydrophobic contributions that supplement the interactions.

**Figure 7:**
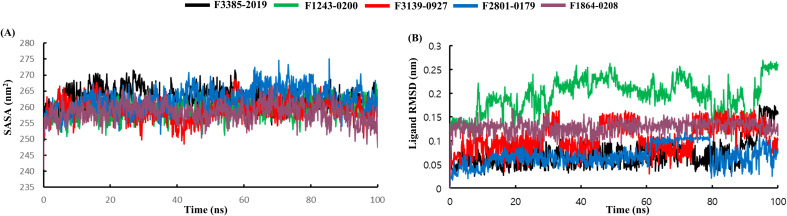
Comprehensive molecular dynamics assessment depicting **(A)** SASA, and **(B)** ligand RMSD fluctuations.

The SASA (**Figure 7A**) showed that all complexes maintained average values between 250 and 270 nm², indicating stable protein surface exposure. F2801–0179 consistently exhibited slightly elevated SASA values, correlating with its increased backbone RMSD and Rg, potentially because of a looser fit or partial exposure of surface residues. In contrast, F1243-0200, F1864-0208, and F3139–0927 displayed more compact profiles with fewer fluctuations, suggesting tighter ligand-induced packing and reduced solvent exposure. Ligand RMSD analysis (**Figure 7B**) was used to assess how well the ligand remained within the protein binding pocket during the simulation. F1243–0200 showed the highest ligand RMSD, reaching ~0.28 nm, suggesting minor reorientation within the pocket, though without dissociation. In contrast, F2801-0179, F3385-2019, and F1864–0208 displayed ligand RMSD values under 0.15 nm, indicating a firm and consistent binding pose. F3139–0927 remained tightly bound as well, reflecting excellent spatial retention.

Collectively, the MD simulation results suggest that F1243–0200 and F1864–0208 form the most stable and well-integrated complexes with GroEL, as evidenced by their low protein RMSD, tight ligand RMSD, stable hydrogen bonding, and compact Rg/SASA profiles. Although F2801–0179 demonstrated increased flexibility and solvent exposure, its stable ligand orientation and moderate H-bonding suggest potential as a flexible binder. These insights complement the docking analysis, highlighting F1243–0200 and F1864–0208 as promising natural inhibitors of GroEL. Notably, the distinct dynamic behavior observed for F2801–0179 across multiple structural metrics suggests a fundamentally different interaction mode compared with the other compounds. The elevated backbone RMSD, increased residue-level fluctuations in flexible loop regions, and higher SASA values indicate that F2801–0179 induces greater conformational adaptability within the GroEL binding pocket. This behavior may arise from differences in its chemical scaffold or suboptimal complementarity with key binding-site residues, resulting in weaker structural anchoring despite maintaining a stable ligand orientation. These observations highlight that increased binding flexibility does not necessarily translate into enhanced complex stability, underscoring the importance of integrating dynamic analyses beyond docking scores alone.

### 3.5 Essential dynamics

**Figure 8:**
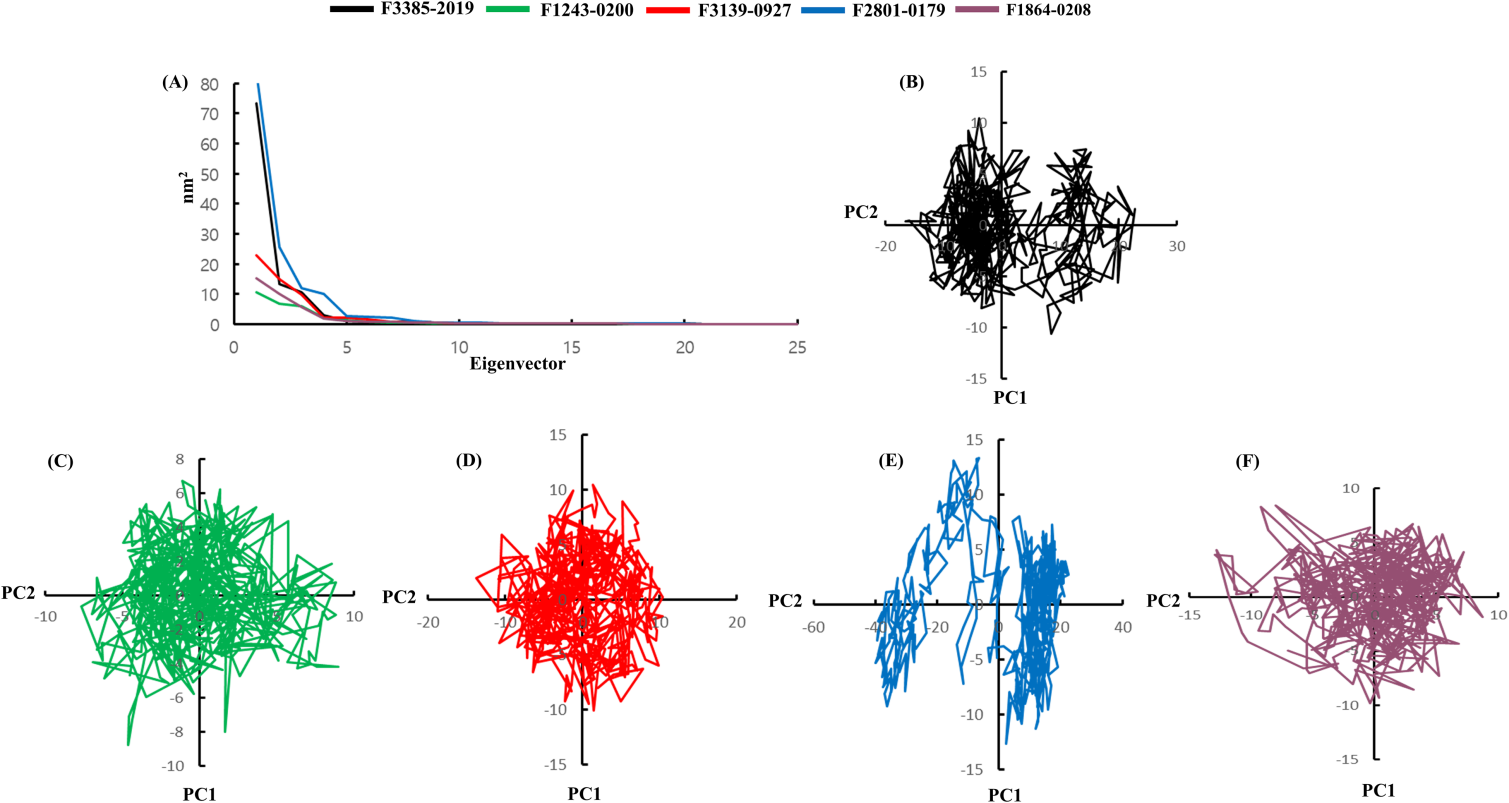
Principal component analysis illustrating conformational sampling of GroEL-ligand complexes. **(A)** Eigenvalue distribution and combined PC1 vs. PC2 scatter plot for all compounds. Individual PCA projections are displayed for: **(B)** F3385-2019 (black), **(C)** F1243-0200 (green), **(D)** F3139-0927 (red), **(E)** F2801-0179 (blue), and **(F)** F1864-0208 (purple).

PCA was performed to investigate the collective atomic motions and conformational transitions of the GroEL protein in complex with each of the five selected natural ligands over the 50 ns of the MDS. PCA reduces the dimensionality of atomic fluctuations and reveals dominant movement patterns by analyzing the eigenvectors derived from the covariance matrix of Cα atomic displacements. The eigenvalue distribution (**Figure 8**) shows that the first three eigenvectors accounted for the majority of the conformational variance in each complex. F2801–0179 exhibited the highest magnitude of motion among the first few principal components, indicating a greater degree of flexibility in the complex. In contrast, F1243–0200 and F1864–0208 displayed lower eigenvalue contributions, suggesting reduced internal motion and higher conformational stability during the simulation. The PC1 versus PC2 projection highlights the spatial distribution of the conformations sampled by each system. The F1243–0200 and F1864–0208 complexes formed tightly clustered groups in essential space, reflecting limited conformational drift and a more compact motion profile. In contrast, F2801–0179 exhibited an extended, scattered trajectory, indicating broader sampling of the conformational space. F3385–2019 and F3139–0927 showed moderate clustering, with some degree of structural flexibility. The individual PC1–PC2 trajectories (**Figure 8**) further visualized the dynamic motion of each complex. The GroEL complex with F1243–0200 followed a tightly grouped trajectory path with minimal variation, whereas F2801–0179 displayed a widespread, irregular motion path, suggesting higher conformational variability. F3139-0927, F3385-2019, and F1864–0208 occupied intermediate spaces, showing moderate fluctuation with overall stable dynamic behavior.

**Figure 9:**
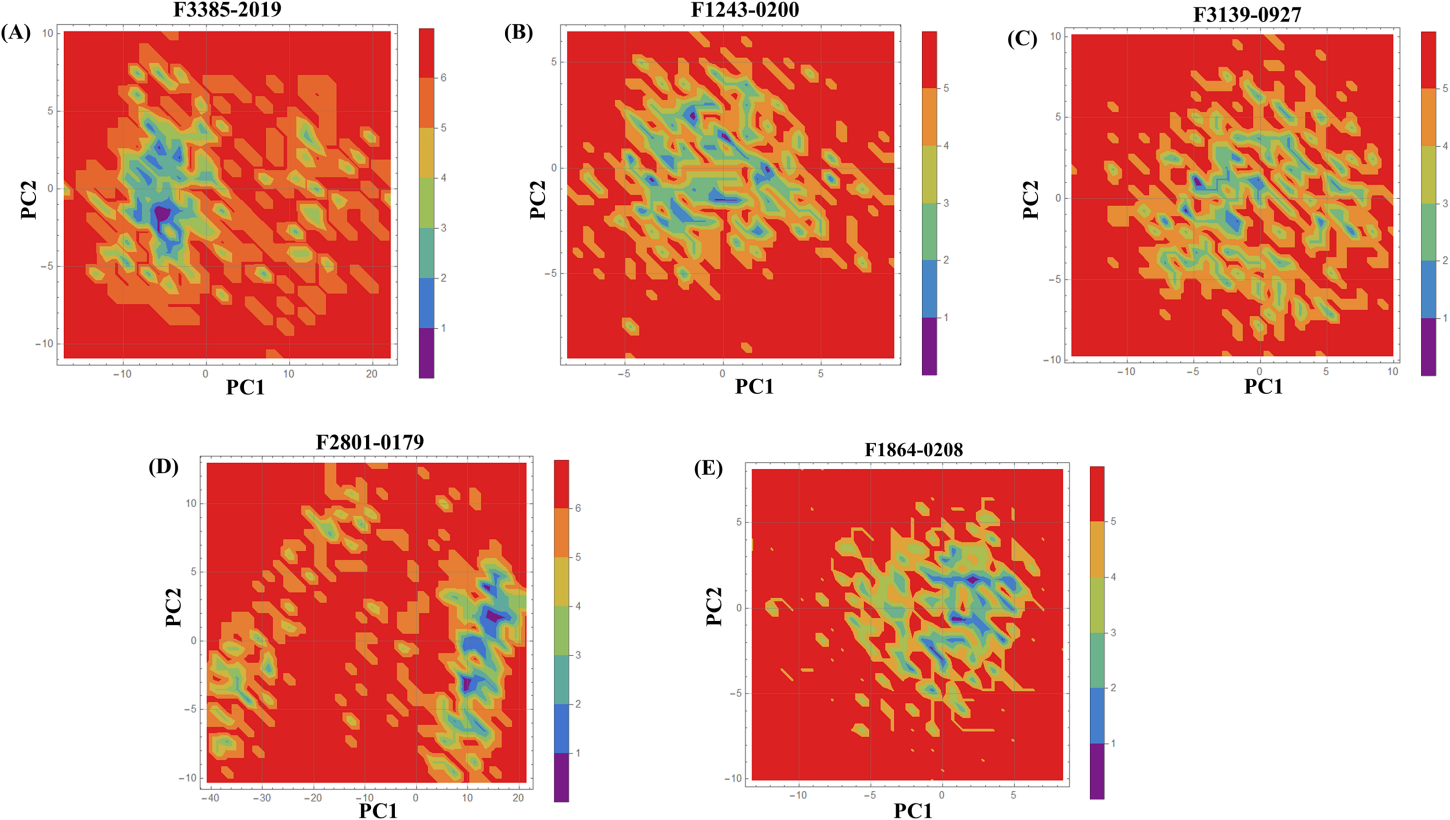
FEL analysis of compounds complexed with GroEL protein. Two-dimensional energy contour maps along principal components PC1 and PC2 for: **(A)** F3385-2019, **(B)** F1243-0200, **(C)** F3139-0927, **(D)** F2801-0179, and **(E)** F1864-0208. Energy values are represented by color gradients from low (blue/purple) to high (red) free energy regions.

To further elucidate the energetic landscape and thermodynamic stability of the GroEL-ligand complexes, FEL analysis was conducted based on the first two principal components (**Figure 9**). The FEL maps provide quantitative insights into the relative stability of different conformational states by visualizing the energy barriers and minima across the sampled conformational space. The energy contour plots revealed distinct patterns for each complex, with color gradients representing free energy variations from stable (blue) to unstable (red) regions. The F1243–0200 complex (**Figure 9B**) displayed well-defined, deep energy minima with narrow basins, indicating highly stable conformational states with limited transitions between energy wells. Similarly, F1864-0208 (**Figure 9E**) exhibited concentrated low-energy regions with sharp energy gradients, thereby reinforcing its conformational stability. In contrast, F2801-0179 (**Figure 9D**) showed a broader and more diffuse energy landscape with multiple shallow minima, which was consistent with the higher flexibility and conformational diversity observed in the PCA analysis. The energy surfaces for F3385-2019 (**Figure 9A**) and F3139-0927 (**Figure 9C**) exhibited intermediate characteristics, featuring moderately defined energy basins with occasional higher-energy transitions. FEL analysis corroborated the PCA findings, demonstrating that F1243–0200 and F1864–0208 occupy the most thermodynamically favorable conformational states with minimal energy barriers for local fluctuations. The deeper energy wells observed for these complexes suggest stronger binding interactions and a reduced likelihood of dissociation. Conversely, the flatter energy landscape of F2801–0179 indicates greater conformational freedom but potentially weaker binding affinity.

Collectively, the PCA and FEL results reinforce the dynamic stability trends observed in the previous RMSD, Rg, and hydrogen bond analyses. The F1243–0200 and F1864–0208 complexes demonstrated the most stable dynamic profiles and favorable energetic landscapes, while F2801–0179 exhibited greater flexibility and energy dispersion, which may correspond to looser binding or adaptability within the GroEL binding site. These findings enhance our understanding of the structural behavior of GroEL-ligand complexes and support the potential of F1243–0200 and F1864–0208 as robust and stable GroEL inhibitors.

### 3.6 MMPBSA analysis

**Figure 10:**
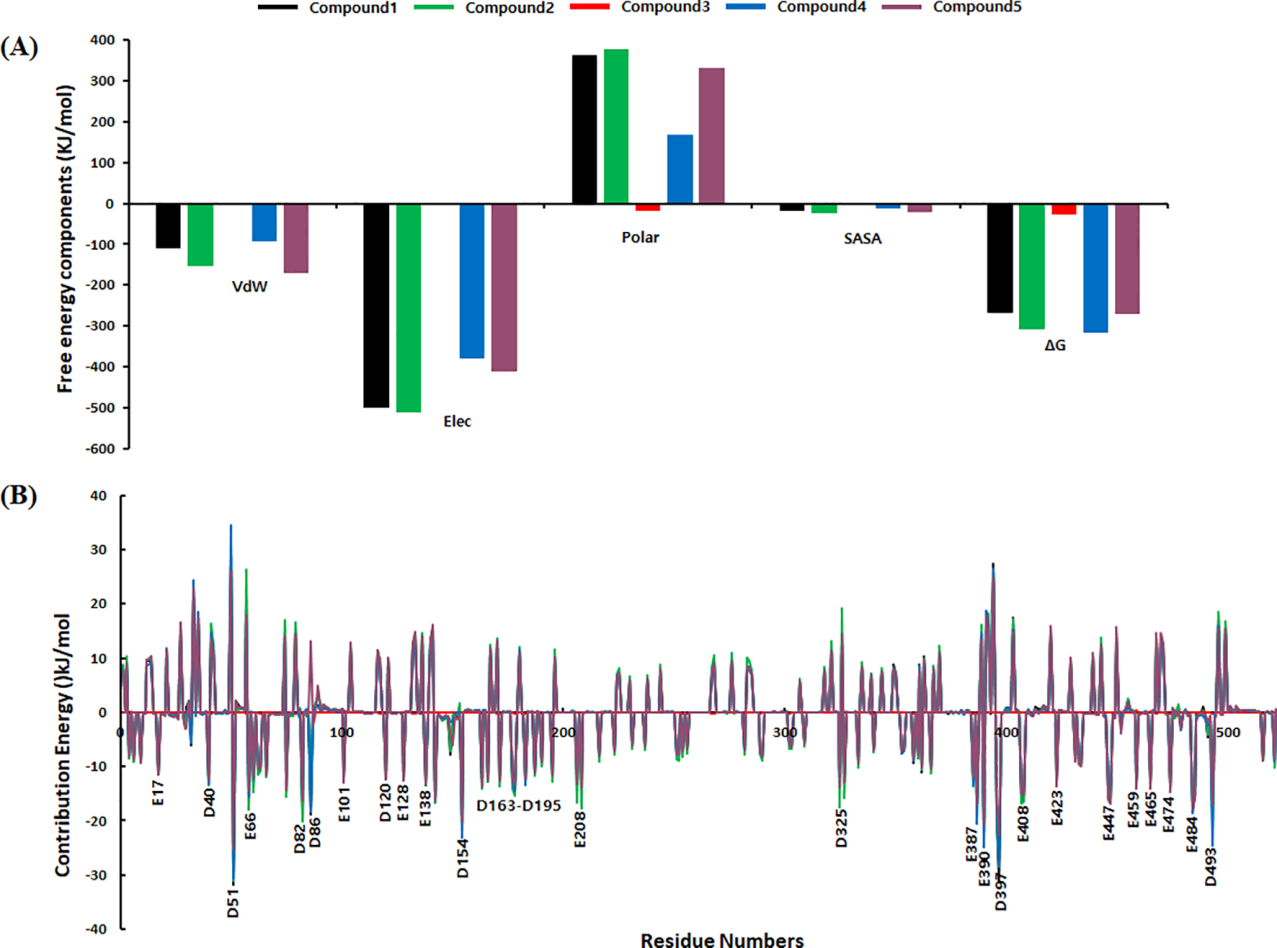
**(A)** Binding Free energy components in KJ/mol. **(B)** Residual energy contributions profile.

To quantitatively assess the binding affinity and thermodynamic favorability of the GroEL-ligand complexes, MMPBSA calculations were performed on the last 50 ns of the MD trajectories. The binding free energies and their individual energy components are summarized in **Table 4**, providing detailed insights into the energetic contributions governing complex stability. MMPBSA analysis revealed significant variations in binding affinities among the five compounds. F2801–0179 demonstrated the most favorable binding energy (-317.677 ± 43.984 kJ/mol), followed closely by F1864-0208 (-269.698 ± 19.107 kJ/mol) and F3385-2019 (-267.150 ± 41.947 kJ/mol). F1243–0200 exhibited a moderately favorable binding energy (-309.769 ± 24.983 kJ/mol), while F3139–0927 showed the least favorable interaction (-26.711 ± 62.260 kJ/mol) (**Figure 10A**). Analysis of the individual energy components provided mechanistic insights into the binding interactions. Van der Waals interactions contributed significantly to complex stabilization across all systems, with F1864–0208 showing the strongest vdW component (-169.804 ± 10.133 kJ/mol), followed by F1243-0200 (-152.576 ± 15.917 kJ/mol) and F3385-2019 (-111.635 ± 16.468 kJ/mol). Electrostatic interactions predominantly displayed unfavorable contributions, likely owing to desolvation penalties upon complex formation. However, F2801–0179 showed a relatively smaller electrostatic penalty (-94.313 ± 43.984 kJ/mol) compared to other compounds. The polar solvation energies were generally unfavorable for most complexes, indicating the disruption of favorable water-protein and water-ligand interactions upon binding. F3139–0927 showed the least unfavorable polar solvation energy (-18.628 ± 67.633 kJ/mol), while F1243–0200 exhibited the most significant penalty (379.061 ± 53.568 kJ/mol). The SASA component contributed favorably to binding in all cases, with F2801–0179 showing the most favorable contribution (-13.273 ± 3.611 kJ/mol). To identify key binding hotspots and understand the molecular basis of ligand recognition, a per-residue energy decomposition analysis was performed (**Figure 10B**). Energy decomposition profiles revealed specific amino acid residues that contribute significantly to ligand binding. Several residues showed consistently favorable interactions across multiple compounds, indicating critical binding determinants within the GroEL active site.

**Table 4:** Binding free energy (BFE) components estimated using the MMPBSA method for all protein–ligand complexes (values, expressed in kJ/mol, provide a detailed measure of the binding affinity of each complex).

Chemical compounds	Van der Waal energy (kJ/mol)	Polar solvation energy (kJ/mol)	SASA energy (kJ/mol)	Binding energy (kJ/mol)
F3385-2019	–111.635 +/– 16.468	363.308 +/– 74.557	–17.017 +/– 1.217	–267.150 +/– 41.947
F1243-0200	–152.576 +/– 15.917	379.061 +/– 53.566	–23.945 +/– 1.908	–309.769 +/– 24.983
F3139-0927	–4.019 +/– 12.940	–18.628 +/– 67.633	–0.723 +/– 2.716	–26.711 +/– 62.260
F2801-0179	–94.313 +/– 26.233	169.037 +/– 111.554	–13.273 +/– 3.611	–317.677 +/– 43.984
F1864-0208	–169.804 +/– 10.133	330.824 +/– 20.474	–20.285 +/– 0.697	–269.698 +/– 19.107

The per-residue analysis identified both stabilizing and destabilizing interactions, with energy contributions ranging from approximately -30 to +30 kJ/mol per residue. In particular, residues E17, D40, D51, E66, D82, D86, E101, D120, E128, E138, D154, D163- D195, E208, D325, E387, E390, D397, E408, E423, E44, E459, E465, E474, E484, and D493 primarily contribute to the binding of these compounds to the GroEL receptor. These residues exhibited strong, favorable interactions (negative energy contributions), which appear to be crucial for maintaining stable ligand binding. The differential per-residue interaction patterns among the five compounds provide insights into the selectivity and specificity of ligand recognition. The combined MMPBSA and per-residue decomposition analyses demonstrate that F2801–0179 and F1864–0208 form the most thermodynamically stable complexes with GroEL, primarily driven by favorable van der Waals interactions and optimized desolvation effects. These findings complement the structural stability observations from the MDS and support the identification of these compounds as promising GroEL modulators. Substantial binding free energies of −269.698 kcal/mol and −267.150 kcal/mol, respectively, further validate their suitability as potential inhibitors of GroEL. In contrast, F3139–0927 showed a significantly weaker binding energy of −26.711 kcal/mol, suggesting lower complex stability during the final simulation window. To contextualize the pharmacological relevance of the identified lead candidates, comparison with established antibacterial agents currently used in clinical practice is informative. Benchmarking docking scores, interaction profiles, and MD-derived stability parameters against reference drugs such as ciprofloxacin, amoxicillin, or gentamicin would allow a clearer assessment of the relative binding strengths and dynamic stability of the proposed compounds, thereby enhancing the translational significance of the present findings.

## 4 Conclusion

This study presents a comprehensive computational framework to identify natural product-based inhibitors targeting the GroEL chaperonin of *L. interrogans*, a critical protein involved in biofilm formation and stress adaptation. Among the screened compounds, F1243–0200 and F1864–0208 emerged as the most promising candidates, exhibiting strong binding affinities, favorable electronic properties, excellent pharmacokinetic profiles, and stable interactions with the GroEL active site in MDS. PCA further confirmed their ability to restrict the conformational flexibility of GroEL, potentially impairing its functional dynamics. These findings suggest that the selective inhibition of GroEL may serve as an effective strategy to disrupt biofilm stability and attenuate leptospiral persistence. Importantly, comparative dynamic and energetic analyses revealed that sustained structural stabilization of GroEL, rather than binding affinity alone, is a key determinant of effective inhibition, underscoring the value of integrating molecular dynamics–based metrics into lead prioritization. This study highlights the potential of natural product scaffolds for anti-leptospiral drug discovery, providing a rational basis for further experimental validation through *in vitro* assays, mutational studies, and structural characterization. Future efforts should focus on biochemical validation of GroEL inhibition, evaluation of antibiofilm efficacy in cellular systems, and *in vivo* assessment of therapeutic potential and safety. In parallel, structure-guided optimization of the identified scaffolds may further enhance potency and selectivity. Integrating cheminformatics with high-throughput screening and *in vivo* models could accelerate the translation of these leads into viable therapeutic candidates.

## Data Availability

The original contributions presented in the study are included in the article. Further inquiries can be directed to the corresponding author.

## References

[B1] Abdeen S. Salim N. Mammadova N. Summers C. M. Frankson R. Ambrose A. J. . (2016). GroEL/ES inhibitors as potential antibiotics. Bioorg Med. Chem. Lett. 26, 3127–3134. doi: 10.1016/j.bmcl.2016.04.089, PMID: 27184767

[B2] Amadei A. Linssen A. B. Berendsen H. J. (1993). Essential dynamics of proteins. Proteins 17, 412–425. doi: 10.1002/prot.340170408, PMID: 8108382

[B3] Amamura T. A. Courrol D. dos S. Barbosa A. S. Silva-Junior I. A. da Silva T. F. . (2025). Proteolytic activity of secreted proteases from pathogenic leptospires and effects on phagocytosis by murine macrophages. Microbes Infection 27, 105469. doi: 10.1016/j.micinf.2025.105469, PMID: 39761846

[B4] Azócar-Aedo L. Monti G. (2022). Seroprevalence of pathogenic Leptospira spp. in domestic dogs from southern Chile and risk factors associated with different environments. Prev. Veterinary Med. 206, 105707. doi: 10.1016/j.prevetmed.2022.105707, PMID: 35835048

[B5] Bekker H. Berendsen H. Dijkstra E. Achterop S. Vondrumen R. Vanderspoel D. . (1993). GROMACS - A PARALLEL COMPUTER FOR MOLECULAR-DYNAMICS SIMULATIONS: 4th international conference on computational physics (PC 92). Phys. Computing 92, 252–256.

[B6] Berendsen H. J. C. Postma J. P. M. van Gunsteren W. F. DiNola A. Haak J. R. (1984). Molecular dynamics with coupling to an external bath. J. Chem. Phys. 81, 3684–3690. doi: 10.1063/1.448118

[B7] Bhatta R. S. Pellicane G. Tsige M. (2015). Tuning range-separated DFT functionals for accurate orbital energy modeling of conjugated molecules. Comput. Theor. Chem. 1070, 14–20. doi: 10.1016/j.comptc.2015.07.022

[B8] Bjelkmar P. Larsson P. Cuendet M. A. Hess B. Lindahl E. (2010). Implementation of the CHARMM force field in GROMACS: analysis of protein stability effects from correction maps, virtual interaction sites, and water models. J. Chem. Theory Comput. 6, 459–466. doi: 10.1021/ct900549r, PMID: 26617301

[B9] Bochevarov A. D. Harder E. Hughes T. F. Greenwood J. R. Braden D. A. Philipp D. M. . (2013). Jaguar: A high-performance quantum chemistry software program with strengths in life and materials sciences. Int. J. Quantum Chem. 113, 2110–2142. doi: 10.1002/qua.24481

[B10] Brylinski M. Skolnick J. (2008). A threading-based method (FINDSITE) for ligand-binding site prediction and functional annotation. Proc. Natl. Acad. Sci. 105, 129–134. doi: 10.1073/pnas.0707684105, PMID: 18165317 PMC2224172

[B11] Capra J. A. Laskowski R. A. Thornton J. M. Singh M. Funkhouser T. A. (2009). Predicting protein ligand binding sites by combining evolutionary sequence conservation and 3D structure. PloS Comput. Biol. 5, e1000585. doi: 10.1371/journal.pcbi.1000585, PMID: 19997483 PMC2777313

[B12] Davignon G. Cagliero J. Guentas L. Bierque E. Genthon P. Gunkel-Grillon P. . (2023). Leptospirosis: toward a better understanding of the environmental lifestyle of Leptospira. Front. Water 5. doi: 10.3389/frwa.2023.1195094

[B13] Davignon G. Pietrosemoli N. Benaroudj N. Soupé-Gilbert M.-E. Cagliero J. Turc É. . (2024). Leptospira interrogans biofilm transcriptome highlights adaption to starvation and general stress while maintaining virulence. NPJ Biofilms Microbiomes 10, 95. doi: 10.1038/s41522-024-00570-0, PMID: 39349472 PMC11442865

[B14] Dias C. S. Pinna M. H. (2025). Leptospira biofilms: implications for survival, transmission, and disease management. Appl. Environ. Microbiol. 91, e01914–e01924. doi: 10.1128/aem.01914-24, PMID: 39791876 PMC11837522

[B15] Douchet L. Goarant C. Mangeas M. Menkes C. Hinjoy S. Herbreteau V. (2022). Unraveling the invisible leptospirosis in mainland Southeast Asia and its fate under climate change. Sci. Total Environ. 832, 155018. doi: 10.1016/j.scitotenv.2022.155018, PMID: 35390383

[B16] Evangelista K. V. Coburn J. (2010). Leptospira as an emerging pathogen: a review of its biology, pathogenesis and host immune responses. Future Microbiol. 5, 1413–1425. doi: 10.2217/fmb.10.102, PMID: 20860485 PMC3037011

[B17] Fayet O. Ziegelhoffer T. Georgopoulos C. (1989). The groES and groEL heat shock gene products of Escherichia coli are essential for bacterial growth at all temperatures. J. Bacteriology 171, 1379–1385. doi: 10.1128/jb.171.3.1379-1385.1989, PMID: 2563997 PMC209756

[B18] Genheden S. Ryde U. (2015). The MM/PBSA and MM/GBSA methods to estimate ligand-binding affinities. Expert Opin. Drug Discov. 10, 449–461. doi: 10.1517/17460441.2015.1032936, PMID: 25835573 PMC4487606

[B19] Gizamba J. M. Mugisha L. (2023). Leptospirosis in humans and selected animals in Sub-Saharan Africa 2014–2022: a systematic review and meta-analysis. BMC Infect. Dis. 23, 649. doi: 10.1186/s12879-023-08574-5, PMID: 37784071 PMC10546638

[B20] Godek J. Sivinski J. Watson E. R. Lebario F. Xu W. Stevens M. . (2024). Bis-sulfonamido-2-phenylbenzoxazoles validate the groES/EL chaperone system as a viable antibiotic target. J. Am. Chem. Soc. 146, 20845–20856. doi: 10.1021/jacs.4c05057, PMID: 39041457

[B21] Guzmán D. A. Diaz E. Sáenz C. Álvarez H. Cueva R. Zapata-Ríos G. . (2023). Domestic dogs in indigenous Amazonian communities: key players in Leptospira cycling and transmission? bioRxiv. 18, e0011671. doi: 10.1101/2023.09.19.558554, PMID: 38568912 PMC10990217

[B22] Ho J. D. Takara L. E. M. Monaris D. Gonçalves A. P. Souza-Filho A. F. de Souza G. O. . (2021). GroEL protein of the Leptospira spp. interacts with host proteins and induces cytokines secretion on macrophages. BMC Microbiol. 21, 99. doi: 10.1186/s12866-021-02162-w, PMID: 33789603 PMC8011160

[B23] Jorgensen W. L. Chandrasekhar J. Madura J. D. Impey R. W. Klein M. L. (1983). Comparison of simple potential functions for simulating liquid water. J. Chem. Phys. 79, 926–935. doi: 10.1063/1.445869

[B24] Jumper J. Evans R. Pritzel A. Green T. Figurnov M. Ronneberger O. . (2021). Highly accurate protein structure prediction with AlphaFold. Nature 596, 583–589. doi: 10.1038/s41586-021-03819-2, PMID: 34265844 PMC8371605

[B25] Kumari R. Kumar R. Lynn A. (2014). g_mmpbsa—A GROMACS tool for high-throughput MM-PBSA calculations. J. Chem. Inf. Model. 54, 1951–1962. doi: 10.1021/ci500020m, PMID: 24850022

[B26] Lee C. Yang W. Parr R. G. (1988). Development of the Colle-Salvetti correlation-energy formula into a functional of the electron density. Phys. Rev. B Condens Matter 37, 785–789. doi: 10.1103/physrevb.37.785, PMID: 9944570

[B27] Lindahl E. Hess B. van der Spoel D. (2001). GROMACS 3.0: a package for molecular simulation and trajectory analysis: a package for molecular simulation and trajectory analysis. J. Mol. Modeling 7, 306–317. doi: 10.1007/s008940100045

[B28] Lipinski C. A. Lombardo F. Dominy B. W. Feeney P. J. (1997). Experimental and computational approaches to estimate solubility and permeability in drug discovery and development settings. Advanced Drug Delivery Rev. 23, 3–25. doi: 10.1016/S0169-409X(96)00423-1, PMID: 11259830

[B29] Lucas D. S. D. Cullen P. A. Lo M. Srikram A. Sermswan R. W. Adler B. (2011). Recombinant LipL32 and LigA from Leptospira are unable to stimulate protective immunity against leptospirosis in the hamster model. Vaccine 29, 3413–3418. doi: 10.1016/j.vaccine.2011.02.084, PMID: 21396409

[B30] Maisuradze G. G. Leitner D. M. (2007). Free energy landscape of a biomolecule in dihedral principal component space: Sampling convergence and correspondence between structures and minima. Proteins: Structure Function Bioinf. 67, 569–578. doi: 10.1002/prot.21344, PMID: 17348026

[B31] Mendu C. Rashid S. A. Azemin W. S. N. A. W. M. Philip N. (2025). Current antibiotics for leptospirosis: Are still effective? Heliyon 11, 2603–2615. doi: 10.1016/j.heliyon.2024.e41239, PMID: 39802004 PMC11720912

[B32] Ngernsombat C. Suriya U. Prattapong P. Verma K. Rungrotmongkol T. Soonkum T. . (2024). Repurposing FDA-approved drugs targeting FZD10 in nasopharyngeal carcinoma: insights from molecular dynamics simulations and experimental validation. Sci. Rep. 14, 31461. doi: 10.1038/s41598-024-82967-7, PMID: 39733096 PMC11682233

[B33] Oselusi S. O. Dube P. Odugbemi A. I. Akinyede K. A. Ilori T. L. Egieyeh E. . (2024). The role and potential of computer-aided drug discovery strategies in the discovery of novel antimicrobials. Comput. Biol. Med. 169, 107927. doi: 10.1016/j.compbiomed.2024.107927, PMID: 38184864

[B34] Parrinello M. Rahman A. (1981). Polymorphic transitions in single crystals: A new molecular dynamics method. J. Appl. Phys. 52, 7182–7190. doi: 10.1063/1.328693

[B35] Pettersen E. F. Goddard T. D. Huang C. C. Couch G. S. Greenblatt D. M. Meng E. C. . (2004). UCSF Chimera–a visualization system for exploratory research and analysis. J. Comput. Chem. 25, 1605–1612. doi: 10.1002/jcc.20084, PMID: 15264254

[B36] Rajapakse S. Fernando N. Dreyfus A. Smith C. Rodrigo C. (2025). Leptospirosis. Nat. Rev. Dis. Primers 11, 32. doi: 10.1038/s41572-025-00614-5, PMID: 40316520

[B37] Roy A. Yang J. Zhang Y. (2012). COFACTOR: an accurate comparative algorithm for structure-based protein function annotation. Nucleic Acids Res. 40, W471–W477. doi: 10.1093/nar/gks372, PMID: 22570420 PMC3394312

[B38] Sadybekov A. V. Katritch V. (2023). Computational approaches streamlining drug discovery. Nature 616, 673–685. doi: 10.1038/s41586-023-05905-z, PMID: 37100941

[B39] Sahoo S. Lee H.-K. Shin D. (2024a). Structure-based virtual screening and molecular dynamics studies to explore potential natural inhibitors against 3C protease of foot-and-mouth disease virus. Front. Vet. Sci. 10. doi: 10.3389/fvets.2023.1340126, PMID: 38298458 PMC10827980

[B40] Sahoo S. Lee H.-K. Shin D. (2025). Elucidating the structural dynamics induced by active site mutations in 3C protease of foot-and-mouth disease virus. PloS One 20, e0321079. doi: 10.1371/journal.pone.0321079, PMID: 40257971 PMC12011219

[B41] Sahoo S. Purohit P. Samantaray S. Meher B. R. (2024b). Identification of antiviral phytocompounds as potential anti-dengue agents against DENV NS2B/NS3 protease: an integrated molecular modelling and dynamics approach. ChemistrySelect 9, e202400384. doi: 10.1002/slct.202400384

[B42] Sahoo S. Son S. Lee H.-K. Lee J.-Y. Gosu V. Shin D. (2023). Impact of nsSNPs in human AIM2 and IFI16 gene: a comprehensive in silico analysis. J. Biomol Struct. Dyn 42, 2603–2615. doi: 10.1080/07391102.2023.2206907, PMID: 37139544

[B43] Samantaray M. Sahoo S. Sahoo D. P. Sethi G. Singh S. Lee H.-K. . (2025). Computational identification of dual COX-1 and NIK inhibitors from marine microalga *Chlorella vulgaris*. J. Genet. Eng. Biotechnol. 23, 100531. doi: 10.1016/j.jgeb.2025.100531, PMID: 40854650 PMC12268566

[B44] Sethi G. Hwang J. H. Krishna R. (2024). Structure based exploration of potential lead molecules against the extracellular cysteine protease (EcpA) of Staphylococcus epidermidis: a therapeutic halt. J. Biomol Struct. Dyn 42, 9167–9183. doi: 10.1080/07391102.2023.2250455, PMID: 37615425

[B45] Singh M. K. Shin Y. Han S. Ha J. Tiwari P. K. Kim S. S. . (2024). Molecular chaperonin HSP60: current understanding and future prospects. Int. J. Mol. Sci. 25, 5483. doi: 10.3390/ijms25105483, PMID: 38791521 PMC11121636

[B46] Taguchi H. Koike-Takeshita A. (2023). *In vivo* client proteins of the chaperonin GroEL-GroES provide insight into the role of chaperones in protein evolution. Front. Mol. Biosci. 10. doi: 10.3389/fmolb.2023.1091677, PMID: 36845542 PMC9950496

[B47] The UniProt Consortium (2017). UniProt: the universal protein knowledgebase. Nucleic Acids Res. 45, D158–D169. doi: 10.1093/nar/gkw1099, PMID: 27899622 PMC5210571

[B48] Tian W. Chen C. Lei X. Zhao J. Liang J. (2018). CASTp 3.0: computed atlas of surface topography of proteins. Nucleic Acids Res. 46, W363–W367. doi: 10.1093/nar/gky473, PMID: 29860391 PMC6031066

[B49] Van Der Spoel D. Lindahl E. Hess B. Groenhof G. Mark A. E. Berendsen H. J. C. (2005). GROMACS: fast, flexible, and free. J. Comput. Chem. 26, 1701–1718. doi: 10.1002/jcc.20291, PMID: 16211538

[B50] Vanommeslaeghe K. Hatcher E. Acharya C. Kundu S. Zhong S. Shim J. . (2010). CHARMM General Force Field (CGenFF): A force field for drug-like molecules compatible with the CHARMM all-atom additive biological force fields. J. Comput. Chem. 31, 671–690. doi: 10.1002/jcc.21367, PMID: 19575467 PMC2888302

[B51] Verma K. Gopikrishnan M. Yadav A. Razack S. A. Gunasekaran K. Bharti P. K. . (2024). Unveiling *Allium sativum* Phytocompounds as New Antileptospiral Agents via a Structural-Based Virtual Screening Approach. ChemistrySelect 9, e202402413. doi: 10.1002/slct.202402413

[B52] Verma K. Patel K. Yadav A. Gopikrishnan M. Sharma R. Mohan M. . (n.d.). Computational drug repositioning for targeting pfEMP1: potential therapeutics for cerebral malaria in plasmodium falciparum. Biotechnol. Appl. Biochem. doi: 10.1002/bab.70040, PMID: 40808285

[B53] Vinod Kumar K. Lall C. Vimal Raj R. Vedhagiri K. Kartick C. Surya P. . (2017). Overexpression of heat shock GroEL stress protein in leptospiral biofilm. Microbial Pathogenesis 102, 8–11. doi: 10.1016/j.micpath.2016.11.010, PMID: 27865827

[B54] Wang Y. Tong Z. Han J. Li C. Chen X. (2025). Exploring novel antibiotics by targeting the groEL/groES chaperonin system. ACS Pharmacol. Transl. Sci. 8, 10–20. doi: 10.1021/acsptsci.4c00397, PMID: 39816798 PMC11729427

[B55] Yang J. Roy A. Zhang Y. (2013). Protein–ligand binding site recognition using complementary binding-specific substructure comparison and sequence profile alignment. Bioinformatics 29, 2588–2595. doi: 10.1093/bioinformatics/btt447, PMID: 23975762 PMC3789548

[B56] Zhang D. Guo F.-B. Li H. (2025). A computer-aided drug repurposing: the antibacterial agents targeting GroEL. Br. J. Pharmacol. doi: 10.1111/bph.70252, PMID: 41239775

